# Use and feasibility of delayed prescribing for respiratory tract infections: A questionnaire survey

**DOI:** 10.1186/1471-2296-12-34

**Published:** 2011-05-18

**Authors:** Sigurd Høye, Jan C Frich, Morten Lindbæk

**Affiliations:** 1 Antibiotic Center for Primary Care, Department of General Practice, Institute of Health and Society, University of Oslo, P.O.Box 1130 Blindern, N-0318 Oslo, Norway; 2 Department of Health Management and Health Economics, Institute of Health and Society, University of Oslo, Norway

## Abstract

**Background:**

Delayed prescribing of antibiotics for respiratory tract infections (RTIs) lowers the amount of antibiotics consumed. Several national treatment guidelines on RTIs recommend the strategy. When advocating treatment innovations, the feasibility and credibility of the innovation must be taken into account. The objective of this study was to explore GPs use and patients uptake of wait-and-see prescriptions for RTIs, and to investigate the feasibility of the strategy from GPs' and patients' perspectives.

**Methods:**

Questionnaire survey among Norwegian GPs issuing and patients receiving a wait-and-see-prescription for RTIs. Patients reported symptoms, confidence and antibiotics consumption, GPs reported diagnoses, reason for issuing a wait-and-see-prescription and their opinion about the method.

**Results:**

304 response pairs from consultations with 49 GPs were received. The patient response rate was 80%. The most common diagnosis for the GPs to issue a wait-and-see prescription was sinusitis (33%) and otitis (21%). 46% of the patients reported to consume the antibiotics. When adjusted for other factors, the diagnosis did not predict antibiotic consumption, but both being 16 years or more (p = 0,006) and reporting to have a fever (p = 0,012) doubled the odds of antibiotic consumption, while feeling very ill more than quadrupled the odds (p = 0,002). In 210 cases (69%), the GP found delayed prescribing a very reasonable strategy, and 270 patients (89%) would prefer to receive a wait-and-see prescription in a similar situation in the future. The GPs found delayed prescribing very reasonable most frequently in cases of sinusitis (79%, p = 0,007) and least frequently in cases of lower RTIs (49%, p = 0,002).

**Conclusion:**

Most patients and GPs are satisfied with the delayed prescribing strategy. The patients' age, symptoms and malaise are more important than the diagnosis in predicting antibiotic consumption. The GP's view of the method as a reasonable approach depends on the patient's diagnosis. In our setting, delayed prescribing seems to be a feasible strategy, especially in cases of sinusitis and otitis. Educational efforts to promote delayed prescribing in similar settings should focus on these diagnoses.

## Background

General practitioners (GPs) issue more than 90% of antibiotic prescriptions in Norway, and about 60% of these are issued for common respiratory tract infections (RTIs) [1]. RTIs are often self-limiting, and antibiotics have a modest role in the treatment of such conditions [2]. Unnecessary use of antibiotics is a global concern, as it leads to antibiotic resistance, adverse drug reactions, and medicalization of self-limiting disease. Antibiotic prescription rates are relatively low in Norway and other Northern European countries [3], but a recent Norwegian prescription study found that there still is room for improvement [4].

Much effort has been put into developing strategies to reduce over-consumption of antibiotics for RTIs in general practice, and randomized controlled trials have provided evidence for delayed prescribing as an effective strategy. Reported pick up rates for wait-and-see prescriptions varies from 24 - 38% (otitis media) [5,6], 31% (sore throat) [7], 20 - 45% (cough) [8,9], to 48% (common cold) [10]. The safety of the method seems to be good, and there is probably no increase in complication rates, but a longer duration of certain symptoms in some studies [11].

It has been argued that delaying antibiotics has little advantage over avoiding them where it is safe to do so [11]. However, the question regarding safety in handling RTIs is not clear cut, and factors like physician insecurity, patient demands and work load lead GPs to prescribe antibiotics without a good medical indication [12]. GPs experience numerous situations where they find delayed prescribing reasonable [13]. Hence, delayed prescribing might have an important place in the management of RTIs [14]. The strategy is recommended in several national treatment guidelines on RTIs in general practice [15-17], and it is part of the intervention in quality improvement studies on appropriate antibiotics prescribing [18-20].

Delayed prescribing is not universally endorsed by GPs [13,21], though patients seem to be confident and satisfied with wait-and-see prescriptions [7,22]. When advocating treatment innovations to improve quality of care, the feasibility and credibility of the innovation must be taken into account [23]. There is a lack of knowledge on if, and in which situations, GPs find delayed prescribing a reasonable approach, and in which situations GPs choose to use the strategy.

The aim of this study is to explore GPs use and patients uptake of wait-and-see prescriptions for RTIs, and to investigate the feasibility of the strategy from GPs' and patients' perspective.

The terms "delayed prescribing" and "wait-and-see prescription" are used synonymously in the literature. In this paper we use "delayed prescribing" for the strategy, and "wait-and-see prescription" denotes the prescription itself.

## Methods

### Subjects and setting

We translated and adopted a questionnaire on patients' response to delayed prescription used in a previous study [22], and developed a questionnaire on GPs reasons for issuing wait-and-see prescriptions.

**Figure 1:**
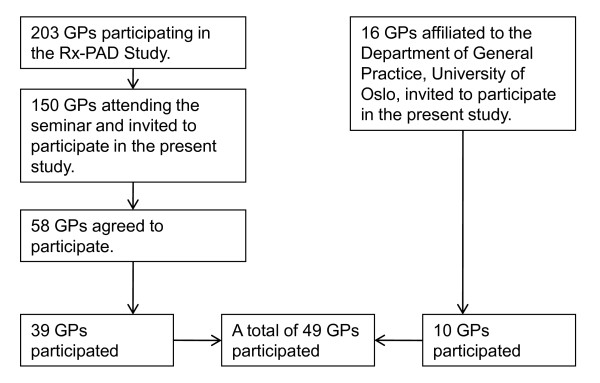
**Flowchart representing GP recruitment**.

The study was conducted as a part of the Prescription Peer Academic Detailing (Rx-PAD) Study, a cluster-randomized educational intervention study in Norwegian general practice with the aim of improving antibiotic prescribing in respiratory tract infections [18]. The elements of the intervention were educational outreach visits to the participants' continuing medical education groups comprising presentation and discussion of evidence-based antibiotics prescribing for RTIs, collection of individual prescription data, audit based on individual feedback reports, as well as a one-day regional seminar. As part of the seminar, one of the authors (SH) gave a lecture on the evidence regarding delayed prescribing, and invited the GPs to recruit patients to the present study. 58 GPs agreed to participate. In addition, 16 GPs affiliated to the Department of General Practice, University of Oslo, were given the same lecture, and agreed to participate (Figure 1).

Eligible patients were those of any age who consulted the GP for a RTI, and to whom the GP found it appropriate to offer a wait-and-see prescription. In the course of the consultation, the GP handed the patient an antibiotic prescription together with a patient questionnaire, a consent form, an information leaflet and a pre-stamped envelope. The patient was instructed to wait for a certain amount of time, chosen by the GP, before deciding whether to take the antibiotics or not. The questionnaire was to be filled once the patient had made this decision. After the consultation, the doctor filled in the GP questionnaire. Patients were rewarded with a scratchcard upon responding, while the GP would receive a gift card for a CD when they had recruited 10 patients. Recruitment took place during April 2006 through June 2008.

The Regional Committee for Research Ethics in Oslo, Norway, approved the study (S-05272).

### Statistical analysis

Chi square test was used to compare those patients who reported to consume antibiotics and those who did not, with regard to both patient factors (demographic characteristics, presenting symptoms, expectations, confidence in deciding whether to use the prescription) and GP factors (diagnose, reason for giving wait-and-see prescription, reasonableness, and impression of expectations and use of the prescription). Logistic regression analysis was performed with the dependent variable being whether the patient reported to consume the antibiotics or not. Further, we compared cases where the GP found delayed prescribing very reasonable and cases where where the GP did not. A significance level of 5% was applied. Analyses were performed using SPSS 14 and 18.

### Material

Out of a total of 68 GPs, 49 (72%) recruited on average 8.5 patients each (median 6; span 1-34). 19 (28%) GPs recruited no patients. We received 413 responses from GPs and 332 responses from patients. Five patients informed that they did not want to participate, and consequently we removed the corresponding GPs responses. For five of the patient responses, we did not receive a corresponding GPs response, resulting in 327 response pairs and a patient response rate of 80%.

17 response pairs were excluded because the GPs had included patients who were treated for other conditions than RTIs, and an additional six response pairs were excluded because the patients failed to answer whether they had taken antibiotics. 304 response pairs remained for analysis.

We grouped diagnoses according to previouos studies on RTIs [1,24]. Table 1 displays the characteristics of the participating patients and GPs.

**Table 1:** Characteristics of participating patients and GPs.

	n	(%)
Patients	304	(100)
Gender		
Female	204	(67)
Male	100	(33)
Grouped age (years)		
Less than 16	100	(33)
16-59	180	(59)
60 and over	24	(8)

GPs	49	(100)
Gender		
Female	13	(27)
Male	36	(73)
Delayed prescriptions issued		
1-4 prescriptions	19	(39)
5-9 prescriptions	12	(24)
10-19 prescriptions	13	(27)
More than 20 prescriptions	5	(10)

## Results

### Comparison of responders and non-responders

Of the 81 non-responding patients, there were significantly more men (47% vs 33%) and more patients with upper RTIs (34% vs 20%), compared to the group of responders.

### Delayed prescribing - when and why

Table 2 shows the diagnoses given by the GPs when issuing a wait-and-see prescription, and the diagnose groups used in the further analysis. In comparison with a reference material of antibiotic prescribing for respiratory tract infections in a Norwegian county during two winter months in 2003, our material shows an overrepresentation of sinusitis (33,2% vs 14,6%) and otitis (21,4% vs 9,1%), and an underrepresentation of lower RTI (13,5% vs 28,5%) and tonsillitis (7,9% vs 16,8%).

**Table 2:** Diagnoses where GPs issued delayed prescription, compared to a reference material of antibiotic prescriptions for respiratory tract infections

						Reference (Vestfold study)	
Diagnose group	Diagnose	ICPC-2 code	n	%	(95% CI)	%	(95% CI)
**Upper respiratory tract symptoms and infections**			**60**	**19,7**	**(15,3-24,2)**	**21,3**	**(20,0-22,6)**
	Cough	r05	6				
	Sinus symptom/complaint	r09	7				
	Throat symptom/complaint	r21	4				
	Upper respiratory infection acute	r74	43				
**Lower respiratory tract infections**			**41**	**13,5**	**(9,7-17,3)**	**28,5**	**(27,1-29,9)**
	Acute bronchitis/bronchiolitis	r78	36				
	Pneumonia	r81	4				
	COPD	r95	1				
**Ear infections**			**65**	**21,4**	**(16,8-26,0)**	**9,1**	**(8,2-10,0)**
	Ear pain/earache	h01	1				
	Ear discharge	h04	1				
	Ear symptom/complaint other	h29	1				
	Acute otitis media/myringitis	h71	62				
**Sinusitis**			**101**	**33,2**	**(27,9-38,5)**	**14,6**	**(13,5-15,7)**
	Sinusitis acute/chronic	r75	101				
**Acute tonsillitis**			**24**	**7,9**	**(4,9-10,9)**	**16,8**	**(15,7-18,0)**
	Strep throat	r72	5				
	Tonsillitis acute	r76	19				
**Other respiratory diagnoses**			**13**	**4,3**	**(2,0-6,6)**	**9,7**	**(8,8-10,6)**
	Laryngitis/tracheitis acute	r77	4				
	Influenza	r80	7				
	Respiratory infection other	r83	2				

The majority (58%) of the children given a wait-and-see-prescription had otitis, while the majority (49%) of adults had sinusitis, and the elderly had lower RTI (46%). Patients with the diagnosis of upper RTI reported feeling more ill (p = 0,009), and patients with tonsillitis felt less ill (p = 0,04) compared to patients with other diagnoses.

The GPs reported that they issued wait-and-see prescriptions mainly because of uncertainty about the indication for antibiotics (211 cases, 69%) or uncertainty about the diagnose (32 cases, 11%). (See also table 4). Difficulties connected to follow up was given as reason in 29 cases (10%), and disagreement with the patient on the need for antibiotics in 12 cases (4%). In 44 cases (14%), the GP reported "Other reasons", and in 34 of these cases, this was the only explanation for issuing the wait-and-see prescription. "Other reasons" were in all but one case described as clinical or therapeutic peculiarities in the specific situation (eg. mild symptoms, pregnancy, short duration of symptoms, other treatment started).

### Factors associated with the decision to consume antibiotics

141 (46%) of the patients reported to consume the antibiotics. Diagnoses and patients' factors associated with consumption of antibiotics are presented in table 3. There were no statistically significant differences between those who reported to have consumed antibiotics and those who did not in respect of their gender or their educational level.

**Table 3:** Patients' characteristics, presenting symptoms and expectations, by consumption of antibiotics.

	Total (%)		Patients who took antibiotics **(%)**^**a **^**(n = 141)**		Patients who did not take **antibiotics (%)**^**a **^ (n = 163)		Pick up rate %	**P-value **^**b**^
Grouped age (years) n = 304								
Less than 16	100	(33)	38	(27)	62	(38)	38	0,04*
16-59	180	(59)	90	(64)	90	(55)	50	0,13
60 and over	24	(8)	13	(9)	11	(7)	54	0,43
Gender n = 304								
Female	204	(67)	95	(67)	109	(67)	47	0,93
Male	100	(33)	46	(33)	54	(33)	46	
Highest education n = 302 (140/162)								
Basic education (7-9 years)	34	(11)	15	(11)	19	(12)	44	0,78
High school (10-12 years)	105	(35)	50	(36)	55	(34)	48	0,75
College/university (>12 years)	163	(54)	75	(53)	88	(54)	46	0,9
Presenting symptoms n = 303 (141/162)								
Sore throat	101	(33)	52	(37)	49	(30)	51	0,21
Earache	94	(31)	40	(28)	54	(33)	43	0,37
Cough	124	(41)	63	(45)	61	(37)	51	0,2
Fever	111	(37)	62	(44)	49	(30)	56	0,012*
Sinus pain	118	(39)	56	(40)	62	(38)	47	0,76
Muscular aches	32	(11)	17	(12)	15	(9)	53	0,42
Runny nose	49	(16)	22	(16)	27	(17)	45	0,82
Nasal congestion	94	(31)	39	(28)	55	(34)	41	0,25
Malaise	72	(24)	38	(27)	34	(21)	53	0,21
Wheezing/shortness of breath	61	(20)	31	(22)	30	(18)	51	0,44
Other symptoms	17	(6)	10	(7)	7	(4)	59	0,29
Sum symptoms	873		430 (3,05 pr case)		443 (2,73 pr case)			
1 symptom	87	(29)	30	(21)	57	(35)	34	0,008*
2-4 symptoms	161	(53)	81	(57)	80	(49)	50	0,15
More than 4 symptoms	55	(18)	30	(21)	25	(15)	55	0,18
Feeling ill n = 301 (139/162)								
Very ill	34	(11)	24	(17)	10	(6)	71	0,003*
Modestly ill	193	(64)	89	(64)	104	(64)	46	0,9
A bit ill	74	(25)	26	(19)	48	(30)	35	0,026*
Patient expectations n = 303 (141/162)								
Antibiotic prescription	157	(52)	79	(56)	78	(48)	50	0,16
Other prescription	44	(15)	20	(14)	24	(15)	45	0,89
Advice	51	(17)	22	(16)	29	(18)	43	0,61
Tests	144	(48)	69	(49)	75	(46)	48	0,61
Referral	9	(3)	3	(2)	6	(4)	33	0,43
Sicknote	43	(14)	24	(17)	19	(12)	56	0,18
No expectations	50	(17)	19	(13)	31	(19)	38	0,19
Diagnosis group n = 304								
Upper respiratory tract symptoms and infections	60	(20)	34	(24)	26	(16)	57	0,075
Lower respiratory tract infections	41	(14)	21	(15)	20	(12)	51	0,5
Ear infections	65	(21)	23	(16)	42	(26)	35	0,045*
Sinusitis	101	(33)	47	(33)	54	(33)	47	0,97
Acute tonsillitis	24	(8)	11	(8)	13	(8)	46	0,96
Other respiratory diagnoses	13	(4)	5	(4)	8	(5)	38	0,56

a Percentages within the brackets are those within the patient group.

b Pearson chi-square.

Patients diagnosed with an ear infection were less likely to consume antibiotics. Patients younger than 16 years were less likely to consume antibiotics (p = 0,04). When reporting to have fever, patients were more likely to consume antibiotics (p = 0,012). Also, a higher number of reported symptoms (p = 0,024) and more malaise (p = 0,012) made patients more likely to consume antibiotics.

The prognostic variables in table 3 resulting in a p-value of 0,25 or less were included in a logistic regression analysis, together with the background characteristics age, gender and educational level, the dependent variable being whether the patient reported to consume the antibiotics or not (Table 4). Symptom sum was not included, as this variable was closely correlated to, and also included, the individual symptoms. Four factors were significantly associated with consuming antibiotics. Having a fever, reporting to be very ill and being of older age increased the odds, while a nasal congestion decreased the odds of consuming antibiotics.

**Table 4:** Logistic multivariate regression analysis

	Unadjusted		**Adjusted **^**a**^	
	Odds ratio (95% CI)	P-value	Odds ratio (95% CI)	P-value
Age (years)				
Less than 16	1		1	
16-60	1,63 (0,99 - 2,69)	0,054	2,21 (1,25 - 3,92)	0,006
60 and over	1,93 (0,79 - 4,74)	0,15	2,89 (1,01 - 8,29)	0,048
Sex				
Male	1		1	
Female	1,02 (0,63 - 1,65)	0,93	0,80 (0,47 - 1,36)	0,41
Highest education				
Basic education (7-9 years)	1		1	
High school (-12 years)	1,15 (0,53 - 2,51)	0,72	1,19 (0,50 - 2,84)	0,7
College/university (>12 years)	1,08 (0,51 - 2,27)	0,84	1,33 (0,58 - 3,05)	0,5
Symptoms ^b^				
Sore throat	1,36 (0,84 - 2,19)	0,21		
Cough	1,35 (0,85 - 2,14)	0,2		
Fever	1,83 (1,14 - 2,93)	0,012	1,94 (1,15 - 3,27)	0,012
Nasal congestion	0,75 (0,46 - 1,23)	0,25	0,58 (0,34 - 0,99)	0,046
Malaise	1,4 (0,82 - 2,38)	0,21		
Feeling ill				
A bit ill	1		1	
Modestly ill	1,58 (0,91 - 2,75)	0,11	1,46 (0,81 - 2,61)	0,21
Very ill	4,43 (1,84 - 10,67)	0,001	4,55 (1,77 - 11,75)	0,002
Patient expectations ^b^				
Antibiotic prescription	1,39 (0,88 - 2,18)	0,16		
Sicknote	1,56 (0,81 - 2,98)	0,18		
No expectations	0,66 (0,36 - 1,24)	0,2		
Diagnosis group				
Sinusitis	1			
Lower respiratory tract infections	1,21 (0,58 - 2,5)	0,61		
Otitis	0,63 (0,33 - 1,2)	0,16		
Upper respiratory tract symptoms and infections	1,5 (0,79 - 2,86)	0,22		
Tonsillitis	0,97 (0,4 - 2,38)	0,95		
Other	0,72 (0,22 - 2,35)	0,58		

Odds ratio for reporting to consume the antibiotics.

a The odds ratios are adjusted for the background characteristics and for the other surviving variables in the model.

b The reference value is *not *having the spesific symptom/expectation.

When asked whether they thought the patient would take the antibiotics, the GPs answered yes in 51 (17%) of the cases, no in 131 (43%) of the cases and that they were uncertain in 122 (40%) of the cases. The GPs' presumption was slightly correlated with the patients' reported action (p = 0,025, correlation coefficient 0,166).

### Feasibility of delayed prescribing

262 (86%) out of the 304 patients stated that they felt confident in deciding whether to use the prescription, 12 patients (4%) felt unconfident, and the remaining 30 patients (10%) felt neither. There were no significant correlations between confidence and certain diagnosis or prescription pick up rate. 270 patients (89%) would prefer to receive a wait-and-see prescription in a similar situation in the future, nine patients (3%) would prefer not to be offered delayed prescribing, whereas 24 patients (8%) were uncertain what they preferred. Patients with upper RTI did to a lesser extent wish for delayed prescribing in the future (48/60, 80%, p = 0,016).

Out of the 163 patients stating not to consume the antibiotics, 64 (39%) reported to have saved the prescription or the medication for later.

In 210 (69%) of the cases, the GPs answered that they viewed delayed prescribing a very reasonable approach in the specific clinical setting. In 90 cases (30%) they found the approach fairly reasonable, and in four cases (1%) they expressed to be uncertain on this subject. Table 5 presents factors associated with GPs finding delayed prescribing a reasonable strategy.

**Table 5:** GPs opinion of delayed prescribing as a reasonable strategy

	Total (%)		Wait-and-see Rx very reasonable (%) (n = 210)		Wait-and-see Rx not very reasonable (%) (n = 94)		Very reasonable %	**P-value **^**a**^
Diagnosis group n = 304								
Upper respiratory tract symptoms and infections	60	(20)	35	(17)	25	(27)	58	0,044*
Lower respiratory tract infections	41	(14)	20	(10)	21	(22)	49	0,002*
Ear infections	65	(21)	50	(24)	15	(16)	77	0,12
Sinusitis	101	(33)	80	(38)	21	(22)	79	0,007*
Acute tonsillitis	24	(8)	17	(8)	7	(7)	71	0,85
Other respiratory diagnoses	13	(4)	8	(4)	5	(5)	62	0,55
GP's reason for giving delayed prescription n = 304								
Uncertainty about indication for antibiotics	211	(69)	151	(72)	60	(64)	72	0,16
Other reason	44	(14)	34	(16)	10	(11)	77	0,2
Uncertainty about diagnose	32	(11)	22	(10)	10	(11)	69	0,97
Difficulties with follow up	29	(10)	19	(9)	10	(11)	66	0,66
Disagreement with the patient	12	(4)	3	(1)	9	(10)	25	0,001*
GP's expectation n = 304								
Patient is likely to take antibiotics	51	(17)	29	(14)	22	(23)	57	0,039*
Patient is not likely to take antibiotics	131	(43)	100	(48)	31	(33)	76	0,017*
Uncertain	122	(40)	81	(39)	41	(44)	66	0,41
GP's impression of patient's antibiotics expectation n = 303 (209/94)								
Patient expected antibiotics	73	(24)	47	(22)	26	(28)	64	0,32
Patient did not expect antibiotics	150	(50)	104	(50)	46	(49)	69	0,93
Uncertain	80	(26)	58	(28)	22	(23)	73	0,44
Grouped age (years) n = 304								
Less than 16	100	(33)	69	(33)	31	(33)	69	
16-59	180	(59)	124	(59)	56	(60)	69	0,98
60 and over	24	(8)	17	(8)	7	(7)	71	
Gender n = 304								
Female	204	(67)	139	(66)	65	(69)	68	0,61
Male	100	(33)	71	(34)	29	(31)	71	

a Pearson chi-square.

12 wait-and-see prescriptions from 10 different GPs were issued because of disagreement with the patient. In three of these cases (25%), the GP found the method very reasonable, as opposed to 71% when the wait-and-see prescription was issued for other reasons.

In sinusitis, the GPs found delayed prescribing very reasonable in 79% of the cases. At the opposite, the GPs found the method very reasonable in 49% of the lower RTI-cases. The GPs found delayed prescribing more reasonable when they thought the patient would not fill in the prescription (p = 0.017).

## Discussion

### Summary of main findings

General practitioners who have been informed about the use of wait-and-see prescriptions in RTIs, most often use the strategy in cases of acute sinusitis and acute otitis media. These are also the diagnoses for which the GPs find the strategy most reasonable. The reported reason for issuing a wait-and-see prescription is most commonly uncertainty about indication for antibiotics.

Patients receiving a wait-and-see prescription are confident in the decision whether to start taking the medication, and half of the patients report to consume the antibiotics. Feeling very ill, having fever, and being more than 16 years predict consumption of antibiotics, while reporting nasal congestion is negatively associated with consuming antibiotics.

### Comparison with existing litterature

To our knowledge, this is the first survey on delayed prescribing in which different diagnoses are compared, and in which the feasability of the strategy among GPs is measured.

We found that GPs issue wait-and-see prescription most commonly in sinusitis and otitis. When compared to a similar group of GPs in Norway [4], our numbers show an over-representation of sinusitis and otitis, which indicates that patients receiving antibiotics for otitis or sinusitis more often will be instructed to wait than patients receiving antibiotics for other conditions. This may be because otitis and sinusitis are the two conditions for which the Norwegian National Treatment Guidelines recommend "watchful waiting" [16]. A Norwegian prescription study shows that tonsilitis is the diagnosis that would most often warrant a prescription for antibiotics, while URTI is at the other extreme [4]. This may explain why patients with tonsilitis in our study felt less ill, and patients with URTI felt more ill, as one could assume that the moderately ill patients with tonsilitis would be given an immediate prescription for antibiotics, and the moderately ill patients with URTI would not be given antibiotics at all.

The first evidence on the advantages of delayed prescribing came from studies on patients with sore throat in the United Kingdom in 1997 [7], and the spreading of this evidence is considered as one of the reasons why antibiotic consumption continued to decrease in the UK from the late 1990s and onwards [25]. However, in our study sore throat is not a condition in which the GPs readily give wait-and-see prescriptions. This may be due to the widespread use of point-of-care streptococcal throat tests in Norwegian general practice [26], and that the GPs let the test results decide whether to prescribe antibiotics.

In our study, 46% of the patients reported to consume the antibiotics and 86% reported confidence in deciding whether to take the antibiotics. These findings are similar to Edwards et al, who in a comparable British study [22] found a consumption rate of 53%, and 87% confident patients. In both studies, fever was found as a predictor for consuming antibiotics. Fever is shown to be the most important cue when parents take treatment decisions on behalf of their sick child [27].

There were some interesting differences regarding patient expectations. Fewer patients in our study expected antibiotics (52%) compared to the findings of Edwards et al (65%). This may be due to a real difference in antibiotic expectation, despite similar antibiotic prescription rates in the two countries [3]. Another explanation may be that the GPs in our study to a lesser degree used delayed prescribing as a tool to meet patient expectation for antibiotics. Substantially more patients in our setting expected tests or referral (50% vs Edwards et al: 2%). This indicates that the more widespread use of point-of-care tests in our setting compared to Edwards et al's UK setting [28] has had an influence on patients' expectations.

We found differences in reported consumption rates for the various diagnoses, and the internal variation shows some resemblence with the results achieved in various diagnose-specific RCTs on delayed prescribing; 35% vs 24 - 38% (otitis media) [5,6], 46% vs 31% (sore throat) [7], 51% vs 20 - 45% (lower RTI/cough) [8,9], and 57% vs 48% (upper RTI/common cold) [10]. The results are understandably not directly comparable, as the methods of issuing delayed prescriptions differ between various studies, the diagnostic criteria varies, and the antibiotic prescription rates [3] and the patients' views on respiratory tract infections show great variance between countries [29]. Nevertheless, the variance between diagnose groups in our study may give valuable information as the prescriptions for various conditions were given in the same setting.

The natural course of otitis in children is a spontaneous recovery after a few days in approximately 80% of the cases [30], whereas other RTIs may not have this sudden relief. This might explain why ear infection is the diagnose with the lowest pick up rate.

The overall satisfaction with delayed prescribing was high both among GPs and patients. GPs consider overuse of antibiotics a problem [31], and may feel uncomfortable prescribing antibiotics [32]. Thus, there is no surprise that GPs in our study found wait-and-see prescriptions most reasonable among patients who they thought would not pick it up.

Although small numbers, our findings suggest that GPs find delayed prescribing more reasonable in situations of clinical uncertainty rather than in situations where patients demand antibiotics, which is in accordance with the findings in a previous, qualitative study among a similar group of GPs [13].

The GPs found delayed prescribing most reasonable in cases of otitis and sinusitis while the strategy was less valued in cases of upper and lower respiratory tract infections. This may also, as suggested above, be due to the difference in the current understanding and recommended treatment of the various conditions; indication for antibiotics in otitis and sinusitis depends partly, according to Norwegian guidelines, on the duration of symptoms. When it comes to bronchitis and URTI/common cold, the main recommendation is to avoid antibiotics altogether. This might explain why these diagnoses were found less appropriate for delayed prescribing.

### Strengths and limitations

The response rate (80%) was relatively high in comparison to a previous study [22]. The aim of this study was not to explore clinical outcomes and safety of the delayed prescribing strategy, and potential differences in treatment outcomes for different diagnoses have not been investigated.

This study does not allow to directly compare the use of wait-and-see prescriptions with the use of prescriptions for antibiotics to be taken immediately, since we have no record of the latter. For illustrative means, we have compared our findings with a reference material of antibiotic prescriptions for RTIs during two winter months.

The participating GPs had agreed to take part in a study on delayed prescribing, and they might hold a more positive view towards the strategy compared to the relatively large group of invited GPs who did not participate. However, both high and low prescribers of wait-and-see-prescriptions were represented.

As in all questionnaire surveys, our results depend on the respondents report, and not necessarily on their action. The patient questionnaire and information leaflet were carefully constructed to avoid an impression that not picking up the prescription would be the preferred solution, so as to minimize a desirability bias. Still, the reported antibiotics consumption rate of 46% may be a underreporting of what actually happened.

The diagnoses referred in this study are the ones chosen by the GPs. We do not know if, and to what extent, diagnostic criteria were followed, and the diagnostic accuracy may have varied between the different GPs.

## Conclusion

Most patients and GPs are satisfied with the delayed prescribing strategy. The patients' age, symptoms and malaise are more important than the diagnosis in predicting antibiotic consumption. The GP's view of the method as a reasonable approach depends on the patient's diagnosis. In our setting, delayed prescribing seems to be a feasible strategy, especially in cases of sinusitis and otitis. Educational efforts to promote delayed prescribing in similar settings should focus on these diagnoses.

## Competing interests

The authors declare that they have no competing interests.

## Authors' contributions

SH and ML conceived and designed the study. SH collected and analysed the data and wrote the draft manuscript. All authors interpreted the data, critically revised the draft for important intellectual content, prepared the manuscript, and gave final approval of the version to be published.

## Authors' information

ML edited and SH took part in developing the Norwegian National guidelines for antibiotic use in primary health care 2008.

## Pre-publication history

The pre-publication history for this paper can be accessed here:

http://www.biomedcentral.com/1471-2296/12/34/prepub

## References

[B1] Straand J Rokstad KS Sandvik H Prescribing systemic antibiotics in general practice. A report from the More & Romsdal Prescription Study Scand J Prim Health Care 1998 16 2 121 127 10.1080/028134398750003296 9689692

[B2] Arroll B Antibiotics for upper respiratory tract infections: an overview of Cochrane reviews Respir Med 2005 99 3 255 261 10.1016/j.rmed.2004.11.004 15733498

[B3] Goossens H Ferech M Vander SR Elseviers M Outpatient antibiotic use in Europe and association with resistance: a cross-national database study Lancet 2005 365 9459 579 587 10.1016/S0140-6736(05)17907-0 15708101

[B4] Gjelstad S Dalen I Lindbaek M GPs' antibiotic prescription patterns for respiratory tract infections - still room for improvement Scand J Prim Health Care 2009 27 4 208 215 10.3109/02813430903438718 PMC3413912 19929185

[B5] Little P Gould C Williamson I Moore M Warner G Dunleavey J Pragmatic randomised controlled trial of two prescribing strategies for childhood acute otitis media BMJ 2001 322 7282 336 342 10.1136/bmj.322.7282.336 PMC26576 11159657

[B6] Spiro DM Tay KY Arnold DH Dziura JD Baker MD Shapiro ED Wait-and-see prescription for the treatment of acute otitis media: a randomized controlled trial JAMA 2006 296 10 1235 1241 10.1001/jama.296.10.1235 16968847

[B7] Little P Williamson I Warner G Gould C Gantley M Kinmonth AL Open randomised trial of prescribing strategies in managing sore throat BMJ 1997 314 7082 722 727 10.1136/bmj.314.7082.722 PMC2126131 9116551

[B8] Dowell J Pitkethly M Bain J Martin S A randomised controlled trial of delayed antibiotic prescribing as a strategy for managing uncomplicated respiratory tract infection in primary care Br J Gen Pract 2001 51 464 200 205 PMC1313951 11255901

[B9] Little P Rumsby K Kelly J Watson L Moore M Warner G Information leaflet and antibiotic prescribing strategies for acute lower respiratory tract infection: a randomized controlled trial JAMA 2005 293 24 3029 3035 10.1001/jama.293.24.3029 15972565

[B10] Arroll B Kenealy T Kerse N Do delayed prescriptions reduce the use of antibiotics for the common cold? A single-blind controlled trial J Fam Pract 2002 51 4 324 328 11978254

[B11] Spurling GK Del Mar CB Dooley L Foxlee R Delayed antibiotics for respiratory infections Cochrane Database Syst Rev 2007 3 CD004417 10.1002/14651858.CD004417.pub3 17636757

[B12] Petursson P GPs' reasons for "non-pharmacological" prescribing of antibiotics. A phenomenological study Scand J Prim Health Care 2005 23 2 120 125 10.1080/02813430510018491 16036552

[B13] Høye S Frich JC Lindbaek M Delayed prescribing for upper respiratory tract infections: a qualitative study of general practitioners' views and experiences Br J Gen Pract 2010 60 581 907 912 10.3399/bjgp10X544087 PMC2991744 21144201

[B14] Little P Delayed prescribing of antibiotics for upper respiratory tract infection BMJ 2005 331 7512 301 302 10.1136/bmj.331.7512.301 PMC1183117 16081428

[B15] Prescribing of antibiotics for self limiting respiratory tract infections in adults and children in primary care 2008 National Institute for Health and Clinical Excellence Clinical Guideline 69 21698847

[B16] The Directorate of Health, The antibiotic center for primary helath care [National guidelines for antibiotic use in primary health care] 2008 Oslo: The Directorate of Health

[B17] STRAMA Medical Products Agency [Diagnostics, treatment and follow-up of acute otitis media (AOM) - new recommendation] Information from the Medical Products Agency 2010 21 5 13 24

[B18] Gjelstad S Fetveit A Straand J Dalen I Rognstad S Lindbaek M Can antibiotic prescriptions in respiratory tract infections be improved? A cluster-randomized educational intervention in general practice--the Prescription Peer Academic Detailing (Rx-PAD) Study [NCT00272155] BMC Health Serv Res 2006 6 75 10.1186/1472-6963-6-75 PMC1569835 16776824

[B19] McDermott L Yardley L Little P Ashworth M Gulliford M Research Team Developing a computer delivered, theory based intervention for guideline implementation in general practice BMC Fam Pract 2010 11 1 90 10.1186/1471-2296-11-90 PMC2995485 21087469

[B20] Simpson SA Butler CC Hood K Cohen D Dunstan F Evans MR Stemming the Tide of Antibiotic Resistance (STAR): a protocol for a trial of a complex intervention addressing the 'why' and 'how' of appropriate antibiotic prescribing in general practice BMC Fam Pract 2009 10 20 10.1186/1471-2296-10-20 PMC2666645 19309493

[B21] Arroll B Goodyear-Smith F Thomas DR Kerse N Delayed antibiotic prescriptions: what are the experiences and attitudes of physicians and patients? J Fam Pract 2002 51 11 954 959 12485551

[B22] Edwards M Dennison J Sedgwick P Patients' responses to delayed antibiotic prescription for acute upper respiratory tract infections Br J Gen Pract 2003 53 496 845 850 PMC1314726 14702903

[B23] Grol R Wensing M What drives change? Barriers to and incentives for achieving evidence-based practice Med J Aust 2004 180 6 Suppl S57 S60 10.5694/j.1326-5377.2004.tb05948.x 15012583

[B24] Gjelstad S Dalen I Lindbaek M GPs' antibiotic prescription patterns for respiratory tract infections--still room for improvement Scand J Prim Health Care 2009 27 4 208 215 10.3109/02813430903438718 PMC3413912 19929185

[B25] Sharland M Kendall H Yeates D Randall A Hughes G Glasziou P Antibiotic prescribing in general practice and hospital admissions for peritonsillar abscess, mastoiditis, and rheumatic fever in children: time trend analysis BMJ 2005 331 7512 328 329 10.1136/bmj.38503.706887.AE1 PMC1183132 15967760

[B26] Fagan M [Is otitis and tonsillitis handled in the same way within normal working hours and out-of-hours?] Tidsskr Nor Laegeforen 2008 128 20 2340 2342 19096491

[B27] Lagerlov P Loeb M Slettevoll J Lingjaerde OC Fetveit A Severity of illness and the use of paracetamol in febrile preschool children; a case simulation study of parents' assessments Fam Pract 2006 23 6 618 623 10.1093/fampra/cml046 17035288

[B28] Butler CC Simpson S Wood F General practitioners' perceptions of introducing near-patient testing for common infections into routine primary care: a qualitative study Scand J Prim Health Care 2008 26 1 17 21 10.1080/02813430701726285 PMC3406622 18297558

[B29] Deschepper R Vander Stichele RH Haaijer-Ruskamp FM Cross-cultural differences in lay attitudes and utilisation of antibiotics in a Belgian and a Dutch city Patient Educ Couns 2002 48 2 161 169 10.1016/s0738-3991(02)00017-4 12401419

[B30] Glasziou PP Del Mar CB Sanders SL Hayem M Antibiotics for acute otitis media in children Cochrane Database Syst Rev 2004 1 CD000219 10.1002/14651858.CD000219.pub2 14973951

[B31] Simpson SA Wood F Butler CC General practitioners' perceptions of antimicrobial resistance: a qualitative study J Antimicrob Chemother 2007 59 2 292 296 10.1093/jac/dkl467 17110392

[B32] Bradley CP Uncomfortable prescribing decisions: a critical incident study BMJ 1992 304 6822 294 296 10.1136/bmj.304.6822.294 PMC1881047 1739831

